# Phytochemical insights into flavonoids in cancer: Mechanisms, therapeutic potential, and the case of quercetin

**DOI:** 10.1016/j.heliyon.2025.e42682

**Published:** 2025-02-13

**Authors:** Piero Alex Silva-Pinto, Janaína Teixeira Costa de Pontes, Brigitte Aguilar-Morón, Christian Shleider Carnero Canales, Fernando Rogério Pavan, Cesar Augusto Roque-Borda

**Affiliations:** a Vicerrectorado de Investigación, Universidad Católica de Santa María de Arequipa, Arequipa, 04000, Republic of Peru; b Department of Biological Sciences, School of Pharmaceutical Sciences, Sao Paulo State University (UNESP), Araraquara, 14800-900, SP, Brazil; c Facultad de Ingeniería de Procesos – Universidad Nacional de San Agustín, Arequipa, Arequipa, Republic of Peru; d Laboratorio BIOMET, Facultad de Ciencias de la Universidad Nacional de Ingeniería, Av. Túpac Amaru 210, Rímac, Lima, Republic of Peru

**Keywords:** Antioxidants, Flavonoids, Quercetin, Signaling pathways, PI3K/Akt/mTOR, NF-κB, JAK/STAT, Nanoformulations, Tumor microenvironment

## Abstract

Quercetin, a flavonoid known for its potent antioxidant and anti-inflammatory properties, has gained attention in cancer therapy due to its ability to modulate key molecular pathways involved in tumor progression and immune evasion. This review provides a comprehensive analysis of quercetin's effects on pathways such as PI3K/Akt/mTOR, MAPK/ERK, NF-κB, and JAK/STAT, which are central to cancer cell survival, proliferation, and apoptosis. Through inhibition of PI3K/Akt/mTOR and MAPK/ERK signaling, quercetin promotes apoptosis and reduces proliferation specifically in cancer cells while sparing healthy cells. Additionally, quercetin downregulates NF-κB activity and modulates JAK/STAT signaling, enhancing immune recognition of cancer cells and decreasing inflammation in the tumor microenvironment. Emerging nanoformulation strategies are also discussed, highlighting how nanotechnology can improve quercetin's bioavailability and targeting capabilities. Unlike other reviews, this work uniquely integrates molecular insights with cutting-edge nanoformulations, showcasing quercetin's dual potential as a therapeutic agent and an immune modulator in the evolving landscape of cancer treatment. This review underscores quercetin's multifaceted role in cancer treatment and suggests future directions to optimize its clinical efficacy, particularly in combination with conventional therapies.

## 1 Introduction

According to the World Health Organization (WHO), it is estimated that in 2024 there will be over 22 million new cases of cancer and around 10.1 million deaths due to neoplasms. The term “cancer” encompasses a variety of diseases (neoplasms) affecting different parts of the body and is characterized by the rapid and uncontrolled proliferation of abnormal cells that invade nearby tissues and spread to other organs (metastasis), which is the leading cause of cancer-related deaths [1]. Risk factors for the development of neoplasms are largely associated with lifestyle, including smoking, alcohol consumption, unbalanced diets, and physical inactivity. Environmental exposures, such as ionizing and UV radiation or carcinogenic chemicals, also contribute significantly to tumour development [2].

Cellular metabolic disruptions caused by these factors lead to oxidative stress, an imbalance between antioxidants and reactive oxygen species (ROS) produced during cellular respiration [3]. ROS, including superoxide radicals, hydrogen peroxide, and hydroxyl radicals, can damage DNA, proteins, and lipids, contributing to genetic mutations and cellular dysfunction [4]. Antioxidants, which mitigate ROS-induced damage, have been extensively studied for their role in preventing and treating diseases linked to oxidative stress [5]. These include both endogenous antioxidants, such as enzymatic and non-enzymatic systems, and exogenous sources, such as vitamins, trace elements, carotenoids, and polyphenols [6]. Among the polyphenols, flavonoids are particularly noteworthy for their antioxidant and anti-cancer properties.

This review specifically focuses on Quercetin (QRT), a flavonoid of significant onco-medical interest due to its potential role in tumour treatment [7,8]. While flavonoids are discussed as a broader family of compounds, QRT serves as the primary subject of analysis because of its extensive therapeutic properties, including its strong antioxidant, anti-inflammatory, and anti-cancer activities. QRT has been widely used in traditional Chinese medicine (TCM) due to its extensive therapeutic properties and it is a key component in various Chinese herbal preparations, such as Huang Qin Tang (*Scutellaria* decoction) [9,10], Xiao Chai Hu Tang (Minor *Bupleurum* decoction) [11], and Ba Zheng San (Eight-herb powder for rectification) [12]. These formulations are traditionally employed for their anti-inflammatory, detoxifying, and immune-modulating effects [13].

In TCM, QRT-containing preparations are believed to regulate the balance of yin and yang, enhance energy flow (qi), and address conditions associated with inflammation, oxidative stress, and metabolic imbalances [14,15]. QRT's role in these formulations often involves synergy with other bioactive compounds to achieve therapeutic efficacy and in modern pharmacological terms, QRT's bioactivities align with its TCM applications [16]. It exhibits strong antioxidant, anti-inflammatory, and anti-cancer properties, supporting its use in preventing and treating conditions associated with oxidative damage and chronic inflammation [17,18]. These dual perspectives, from both traditional and modern medicine, underline the significance of QRT in therapeutic interventions. This review will further analyze its therapeutic efficacy in TCM preparations and explore its potential integration into contemporary oncological treatments.

## 2 Immuno-oncology

Immuno-oncology builds on the premise that the immune system can recognize and eliminate tumor cells, and that certain natural compounds—such as flavonoids—can modulate this process by enhancing or restoring antitumor activity. Among this group of compounds, QRT stands out for its involvement in multiple signaling pathways associated with proliferation, apoptosis, and immune responses against cancer [19,20]. Several highly prevalent tumor types worldwide, particularly colorectal, breast, prostate, and hepatocellular cancers, exhibit alterations in critical regulatory mechanisms that affect both cell growth and immunomodulation. In the case of colorectal cancer, for example, lifestyle habits such as smoking, a high-calorie diet, alcohol consumption, physical inactivity, and obesity significantly contribute to its incidence [21]. These factors, in conjunction with the interplay between the gut microbiota and oxidative stress, underscore the potential role of flavonoids (including QRT) in preventing and controlling tumor progression [22].

Meanwhile, breast cancer, which stands as the second leading cause of cancer-related death among women (after lung cancer) [23], is likewise influenced by lifestyle choices, with obesity, physical inactivity, and smoking or alcohol intake heightening its risk [24,25]. Research indicates that QRT and other flavonoids can modulate key cellular pathways involved in proliferation, apoptosis, and angiogenesis, opening up possibilities for therapeutic and preventive interventions [26]. Similar beneficial effects have been reported for prostate cancer, a major contributor to cancer mortality in men [27]. In this malignancy, inflammation and dysregulated signaling—particularly through pathways like Wnt/β-catenin—play a crucial role in tumorigenesis and metastasis [28].

Notably, certain flavonoids appear capable of modulating proinflammatory mediators such as IL-6, IL-8, and TNF-α, suggesting a protective or synergistic effect when combined with established antitumor therapies. Likewise, hepatocellular carcinoma (e.g., the HepG2 cell model) involves mechanisms like the Warburg effect, which facilitates rapid tumor proliferation under hypoxic conditions [29]. Here again, oxidative stress and metabolic dysregulation serve as potential targets for the antioxidant and cytotoxic properties of flavonoids. QRT and other flavonoids have demonstrated the capacity to modulate essential checkpoint proteins (e.g., p53, p21, p27) and key signaling pathways (e.g., mTOR, PI3K/Akt), which govern cancer cell survival and propagation [30]. In particular, hyperactivation of PI3K/Akt/mTOR contributes to the progression of numerous solid tumors, and flavonoids can inhibit critical kinases within this pathway, thus curtailing tumor growth and inducing apoptosis [31].

## 3 Flavonoids: structure and anticancer activity

**Fig. 1:**
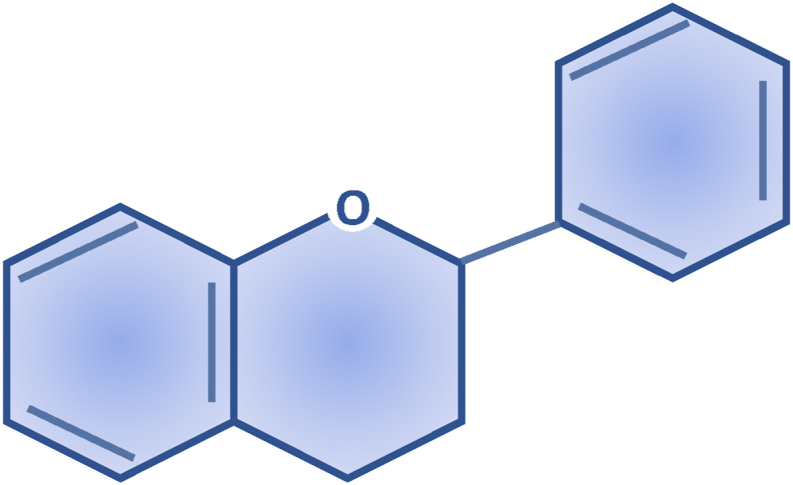
Phenolic rings of the basic structure of flavonoids. Image designed and adapted from Kopustinskiene et al. [34].

Flavonoids are polyphenolic compounds produced by plants as secondary metabolites and play crucial roles in biological processes such as development, growth, and resistance to biotic and abiotic stresses. Additionally, they exhibit multiple health benefits, including antioxidant, anti-inflammatory, and anti-aging properties [32]. Flavonoids have in their chemical structure 3 phenolic rings responsible for their antioxidant function, represented in Fig. 1. These molecules possess its antioxidant mechanism based on the capture of free radicals by donating hydrogen atoms or electrons to neutralize reactive oxygen species (ROS). Additionally, flavonoids exhibit chelation properties, binding to transition metals that can catalyse ROS-generating reactions. They are also capable of inhibiting enzymes involved in ROS production (such as xanthine and oxidase) and restoring other antioxidants (such as vitamins C and E) [32,33]. In addition to the previously mentioned properties, flavonoids may exhibit inhibitory effects on cell proliferation and activate apoptotic pathways in neoplastic cells, making these substances promising for the treatment of certain types of cancer [33]. The main flavonoids in this group are described in Table 1, highlighting their structure, primary antioxidant biological activity, and anticancer effects reported in recent years.

**Table 1:**
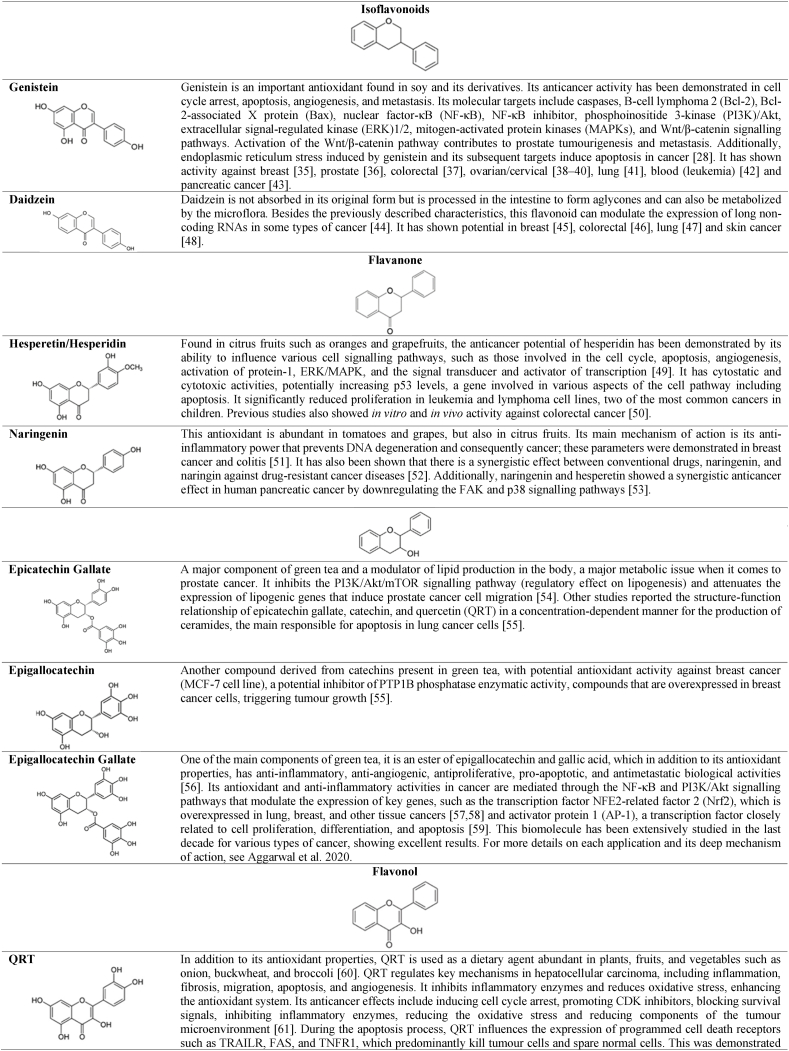

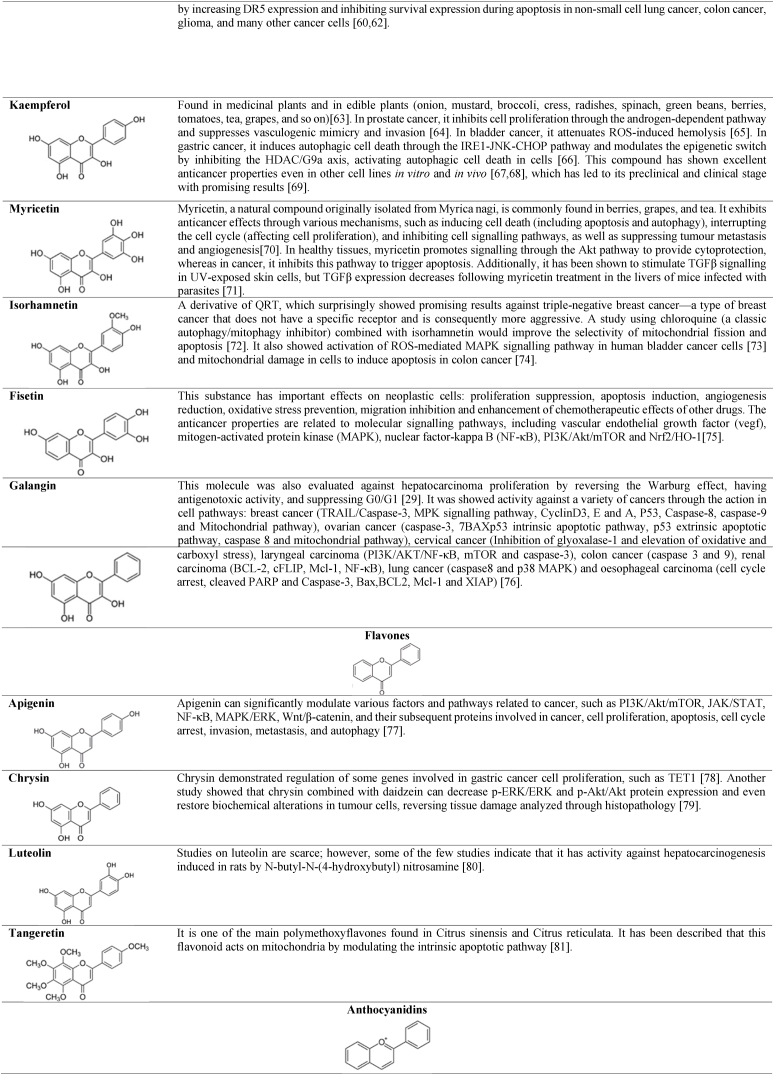

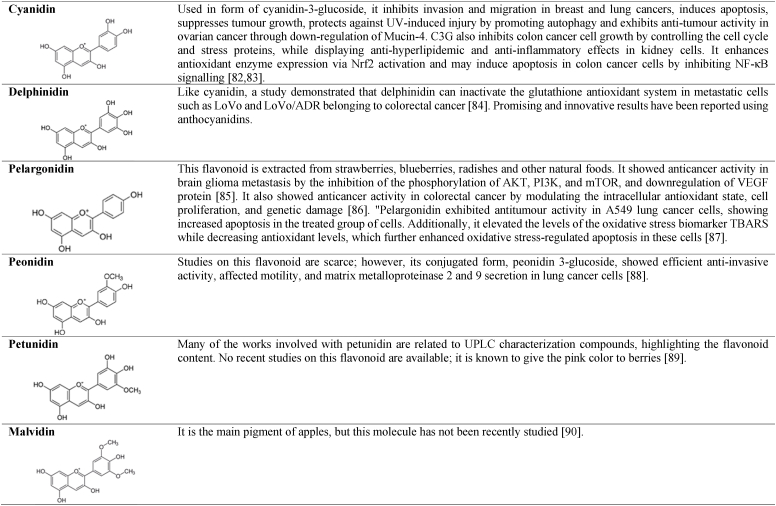
Antioxidant compounds derived from flavonoids, anticancer activity. Images designed in ChemCAD 7 [[35], [36], [37], [38], [39], [40], [41], [42], [43], [44], [45], [46], [47], [48], [49], [50], [51], [52], [53], [54], [55], [56], [57], [58], [59], [60], [61], [62], [63], [64], [65], [66], [67], [68], [69], [70], [71], [72], [73], [74], [75], [76], [77], [78], [79], [80], [81], [82], [83], [84], [85], [86], [87], [88], [89], [90]].

## 4 Quercetin, promising antioxidant in the cancer treatment?

QRT is a naturally occurring flavonoid ubiquitously present in various fruits, vegetables, and grains. Renowned for its potent antioxidant and anti-inflammatory properties, QRT has garnered significant attention in biomedical research [17,91]. Its multifaceted biological activities suggest potential therapeutic applications across a spectrum of diseases. This analysis provides a detailed examination of QRT's strengths, weaknesses, opportunities, and threats (SWOT) within the context of advanced scientific understanding.

***Strengths -*** QRT exhibits strong free radical scavenging abilities, mitigating oxidative stress by neutralizing ROS. This antioxidant capacity is crucial in preventing cellular damage linked to aging and various pathologies, including neurodegenerative diseases and cardiovascular disorders [92]. Furthermore, by inhibiting pro-inflammatory enzymes such as cyclooxygenase and lipoxygenase, QRT modulates inflammatory pathways [93]. It downregulates the expression of cytokines and adhesion molecules, thereby attenuating inflammatory responses central to chronic conditions like arthritis and asthma [94]. Additionally, QRT has demonstrated inhibitory effects on viral replication in multiple studies, suggesting utility against viruses like influenza and hepatitis [95]. It induces apoptosis and inhibits proliferation in various cancer cell lines, indicating potential as an adjunct in oncological therapies [96].

The abundant presence of QRT in common dietary sources such as apples, onions, and berries facilitates its regular consumption [97]. This accessibility supports its use as a dietary supplement and enhances public acceptance due to its natural origin. Moreover, generally recognized as safe (GRAS) by regulatory agencies, QRT exhibits low toxicity in humans at standard dosages. Its safety profile facilitates research and potential therapeutic applications without significant risk of adverse effects [98].

***Weaknesses -*** QRT suffers from low oral bioavailability due to poor water solubility and extensive first-pass metabolism, which reduces its systemic absorption and therapeutic efficacy when administered orally [99]. Furthermore, the compound undergoes rapid phase II metabolism, leading to conjugation and swift excretion and this pharmacokinetic profile necessitates higher or more frequent dosing to achieve therapeutic plasma concentrations, which may not be practical or economical [100]. Additionally, variability in QRT content among dietary sources and supplements leads to inconsistent dosing [101]. Moreover, QRT can modulate the activity of cytochrome P450 enzymes and drug transporters like P-glycoprotein and these interactions may alter the pharmacokinetics of concomitant medications, posing risks of adverse effects or reduced efficacy [102].

***Opportunities -*** Innovative delivery strategies, such as nanoencapsulation and liposomal formulations, can enhance QRT's bioavailability, offering opportunities to overcome pharmacokinetic barriers and improve therapeutic outcomes [[103], [104], [105]]. Furthermore, the growing market for health-promoting foods presents avenues for incorporating QRT into functional products, aligning with consumer trends favouring natural ingredients and preventive healthcare [104]. Additionally, exploring QRT in combination with other bioactive compounds may yield synergistic effects, potentially potentiating therapeutic benefits and opening new frontiers in treating complex diseases [106]. Moreover, a heightened public focus on wellness and natural therapies enhances the demand for products like QRT. Capitalizing on this trend can expand market reach and support further research investments. QRT's multifaceted biological activities also position it as a candidate for managing chronic conditions such as diabetes, hypertension, and metabolic syndrome. Continued research could establish it as a valuable component in comprehensive treatment strategies.

***Threats -*** Navigating the complex regulatory landscape for nutraceuticals and supplements poses significant challenges; stricter regulations may limit market access or require extensive validation, thereby increasing development costs [107]. Moreover, other natural antioxidants and flavonoids, such as resveratrol and catechins, compete within the same market space and their superior efficacy or better bioavailability could overshadow QRT's potential. Although QRT is generally safe, high doses may lead to nephrotoxicity or interfere with thyroid function. Such risks necessitate caution and could deter consumer use if not properly managed [108]. Additionally, overstated health claims without sufficient scientific backing can lead to consumer mistrust, making it essential to ensure accurate representation of QRT's benefits to maintain credibility and market stability. Furthermore, the natural origin of QRT complicates patent protection, potentially limiting commercial incentives [109].

## 5 Quercetin: Molecular mechanism and key strategies

### 5.1 Inhibition of the PI3K/Akt/mTOR pathway

The PI3K/Akt/mTOR pathway is essential in regulating cellular growth, proliferation, migration, and survival, with hyperactivation often observed in various cancers, including breast, lung, and prostate cancer [110]. QRT's inhibitory effect on the PI3K/Akt/mTOR pathway primarily involves the reduction of Akt phosphorylation, which consequently decreases mTOR activity, leading to lower cellular proliferation and increased apoptosis in cancer cells. As mTOR regulates critical processes like protein synthesis and angiogenesis—both vital for tumour growth—its inhibition by QRT effectively hampers tumour progression [20]. In breast cancer models, QRT was shown to suppress phosphorylation within this pathway, thereby promoting cell death in cancer cells while exerting minimal effects on healthy cells. Additionally, by inhibiting this pathway, QRT enhances cancer cell sensitivity to conventional chemotherapies, potentially reducing drug resistance [111].

### 5.2 Modulation of oxidative stress and antioxidant systems

QRT plays a key role in modulating oxidative stress, which is a central factor in cancer progression due to its damaging effects on DNA, proteins, and cellular membranes and it can directly neutralize ROS by donating electrons or hydrogen atoms, effectively reducing cellular oxidative stress [112]. This activity helps protect cells from oxidative damage that may otherwise lead to carcinogenesis [113]. Beyond direct ROS scavenging, QRT also supports cellular antioxidant systems by restoring levels of endogenous antioxidants, such as vitamins C and E and these antioxidants work synergistically to stabilize cellular membranes and maintain cellular integrity [114]. QRT's metal-chelating properties further enhance its antioxidant action by binding to transition metals that catalyse ROS-generating reactions, thus limiting additional ROS production within the tumour microenvironment [115]. This comprehensive antioxidant action not only limits the initiation and progression of tumors but also protects non-cancerous cells from oxidative damage during treatment.

### 5.3 Apoptosis induction and anti-inflammatory activity

QRT exerts significant anticancer effects by inducing apoptosis in tumour cells while simultaneously exhibiting anti-inflammatory properties that help modulate the tumour microenvironment [96]. Through various mechanisms, QRT promotes programmed cell death specifically in cancer cells, which reduces the risk of harm to healthy cells [20]. Key to this process is the activation of death receptors, such as FAS and TNFR1, and the upregulation of caspases, a family of proteases crucial for apoptosis [19]. Studies have shown that QRT increases the expression of pro-apoptotic proteins like Bax while downregulating anti-apoptotic proteins, such as Bcl-2, thus tipping the balance in favour of cell death in cancer cells [116]. In addition to inducing apoptosis, QRT also modulates inflammatory pathways, primarily by inhibiting transcription factors like NF-κB and COX-2, both of which play a role in sustaining chronic inflammation in the tumour microenvironment and by reducing the activity of these inflammatory mediators, QRT mitigates pro-inflammatory cytokine production, thereby limiting the immune evasion and metastatic potential of cancer cells [117].

### 5.4 Modulation of the JAK/STAT pathway

QRT has been shown to modulate the JAK/STAT pathway, which plays a pivotal role in immune response regulation and is often hyperactivated in various cancers, particularly in breast and hematologic malignancies and by inhibiting key components of this pathway, QRT can interfere with signals that promote cancer cell survival, proliferation, and immune evasion [118]. Specifically, QRT suppresses the phosphorylation of STAT proteins, preventing their activation and nuclear translocation and this inhibition disrupts the transcription of genes involved in cell growth and immune suppression, ultimately hindering tumour progression [20]. The impact of QRT on the JAK/STAT pathway is especially relevant in immuno-oncology, as this pathway is a common target for cancer cells attempting to evade immune detection and by inhibiting JAK/STAT signalling, QRT helps to restore immune surveillance, facilitating the recognition and elimination of cancer cells by T cells [119].

### 5.5 Inhibition of the Wnt/β-catenin pathway

The Wnt/β-catenin pathway is a critical regulator of cell proliferation, differentiation, and apoptosis, and its dysregulation is commonly implicated in various cancers, especially colorectal cancer [120]. When hyperactivated, this pathway contributes to uncontrolled cell growth and tumour progression by enabling β-catenin accumulation in the cytoplasm and subsequent translocation to the nucleus, where it drives the transcription of oncogenes such as c-Myc and Cyclin D1 [121]. QRT has demonstrated the ability to interfere with this pathway by reducing β-catenin nuclear accumulation, thereby limiting the expression of these proliferative genes [122]. Studies have shown that QRT effectively downregulates Wnt pathway components, including β-catenin and Dishevelled (DVL) proteins, which are critical for signal propagation within the Wnt cascade and this inhibition reduces cellular proliferation and induces apoptosis in cancer cells, highlighting QRT's potential in cancers characterized by Wnt/β-catenin dysregulation [120].

### 5.6 Inhibition of the MAPK/ERK pathway

The MAPK/ERK (Mitogen-Activated Protein Kinase/Extracellular Signal-Regulated Kinase) pathway is a central signalling cascade that regulates cell growth, survival, and differentiation and aberrant activation of this pathway is frequently observed in various cancers, including melanoma, lung, and breast cancers, where it drives uncontrolled cell proliferation and contributes to resistance against apoptosis [123]. QRT has been shown to block the MAPK/ERK pathway by inhibiting the phosphorylation of ERK, which is necessary for its activation and subsequent translocation to the nucleus and once in the nucleus, activated ERK promotes the expression of genes involved in cell division and survival [124]. By reducing ERK activation, QRT decreases the transcription of these oncogenic targets, leading to reduced cellular proliferation and increased apoptosis in cancer cells. Studies in lung cancer cell lines, for instance, indicate that QRT treatment effectively reduces ERK phosphorylation, impairing cell cycle progression and promoting apoptotic cell death and the inhibition of the MAPK/ERK pathway by QRT can enhance the efficacy of other anticancer treatments [125]. Cancer cells often rely on MAPK/ERK signalling for survival, especially under the stress induced by chemotherapy and radiation and by suppressing this pathway, QRT sensitizes tumour cells to these conventional treatments, potentially improving therapeutic outcomes [[206], [207]]. This dual action of direct tumour inhibition and sensitization to other therapies highlights QRT's potential in combination therapies targeting MAPK/ERK-dependent cancers.

### 5.7 Modulation of the NF-κB pathway

The NF-κB (Nuclear Factor kappa-light-chain-enhancer of activated B cells) pathway is a key regulatory pathway involved in inflammatory responses, cell survival, and immune modulation. In cancer, NF-κB is frequently overactivated, driving the transcription of genes that promote cell survival, proliferation, and resistance to apoptosis [127]. Persistent activation of this pathway also leads to the secretion of pro-inflammatory cytokines that contribute to a tumour-supportive microenvironment, fostering cancer progression and metastasis [128]. QRT has been shown to downregulate the NF-κB pathway, thereby inhibiting the expression of anti-apoptotic and pro-inflammatory genes in cancer cells and this inhibition occurs primarily through the suppression of IκB kinase (IKK), which prevents the phosphorylation and degradation of IκB, the inhibitor of NF-κB [129]. As a result, NF-κB remains sequestered in the cytoplasm, reducing its nuclear translocation and the transcription of target genes involved in cell survival and inflammation. Studies indicate that QRT treatment decreases NF-κB activity in various cancer types, including breast and prostate cancers, thereby sensitizing tumour cells to apoptosis and limiting inflammation-driven tumour growth and by modulating NF-κB signalling, QRT not only induces apoptosis but also creates a less favorable microenvironment for tumour growth [130].

### 5.8 MicroRNA (miRNA) modulation in cancer therapy

MicroRNAs (miRNAs) are a class of small, non-coding RNA molecules that play a significant role in regulating gene expression at the post-transcriptional level [131,132]. They are critically involved in key processes such as cell proliferation, apoptosis, angiogenesis, and metastasis—hallmarks of oncogenesis [133]. Aberrant expression of miRNAs is a common feature of cancer, with some miRNAs acting as oncogenes (oncomiRs) and others as tumor suppressors [134]. QRT, a plant-derived flavonoid, has been identified as a potent modulator of miRNAs, revealing an epigenetic dimension to its anticancer properties [135]. QRT has been shown to inhibit oncomiRs—miRNAs that promote cancer by targeting tumor suppressor genes. For instance.

• miR-21 [136]: A highly upregulated oncomiR in numerous cancers, miR-21 facilitates tumor progression and apoptosis evasion by targeting tumor suppressor genes like PTEN. QRT reduces miR-21 expression, reactivating PTEN activity and downregulating the PI3K/Akt/mTOR pathway, which leads to diminished cell proliferation and increased apoptosis[NO_PRINTED_FORM].
• miR-155 [137]: Associated with inflammation and immune evasion in the tumor microenvironment, miR-155 is another target of QRT. By suppressing miR-155, QRT mitigates inflammation, improves immune response, and reduces metastatic potential.

QRT also enhances the expression of tumor suppressor miRNAs, which suppress oncogenic pathways and encourage apoptosis.

• miR-34a [138]: Known for its role in inducing apoptosis and inhibiting metastasis, miR-34a targets genes like Bcl-2 and SIRT1. QRT upregulates miR-34a, shifting cellular signaling toward pro-apoptotic pathways, thus promoting cancer cell death.
• Let-7 family [139]: QRT increases the expression of Let-7 miRNAs, which suppress oncogenes such as KRAS and HMGA2, reducing tumor growth and progression.

**Fig. 2:**
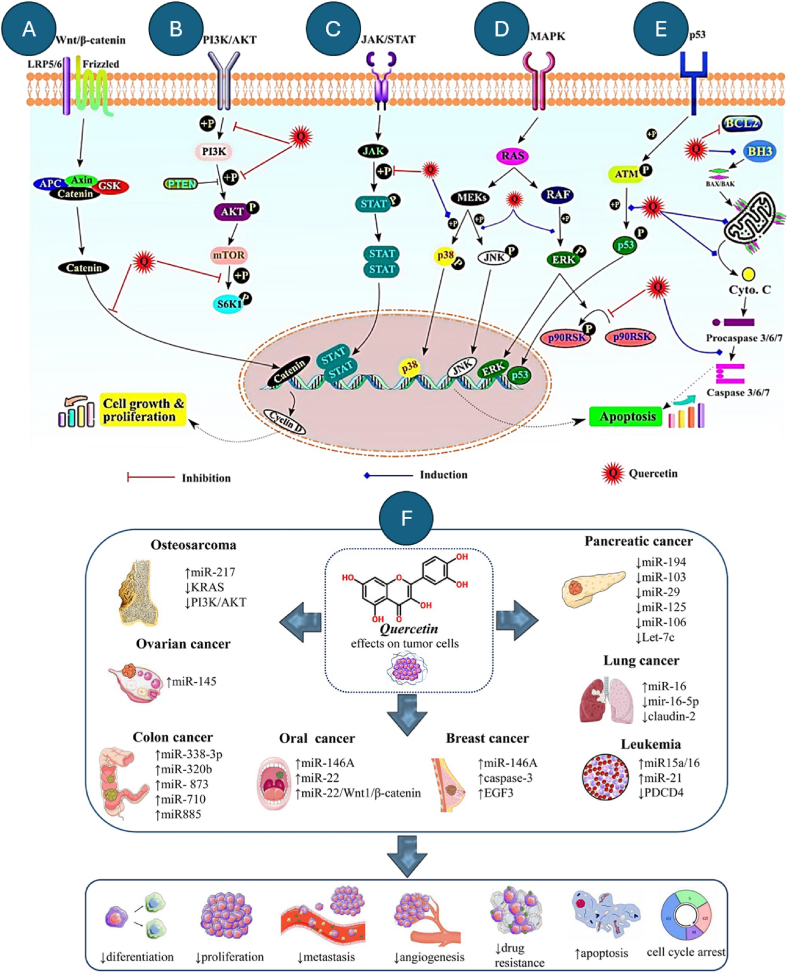
Key signaling pathways modulated by QRT during cancer prevention and its regulation of miRNAs across various cancer types. (A) Wnt/β-catenin pathway: QRT inhibits β-catenin nuclear translocation. (B) PI3K/Akt pathway: QRT blocks phosphorylation of PI3K, Akt, and S6K. (C) JAK/STAT pathway: QRT suppresses phosphorylation of STAT proteins. (D) MAPK pathway: QRT induces phosphorylation of p38, JNK, and ERK. (E) p53 pathway: QRT promotes phosphorylation of p53, activating apoptosis. (F) QRT's regulation of miRNAs (↑ increased, ↓ decreased) and its therapeutic effects on various cancer types, including osteosarcoma, ovarian, colon, oral, pancreatic, lung, breast cancer, and leukemia. Effects include reduced differentiation, proliferation, metastasis, angiogenesis, and drug resistance, while promoting apoptosis and cell cycle arrest. Reprinted/adapted with permission from Asgharian et al. [142]. Copyright 2022, Cancer Cell International from BMC - part of Springer Nature (Open Access).

QRT's dual molecular and epigenetic actions as a modulator of miRNAs present a compelling avenue for advancing cancer therapy. Its ability to influence miRNA expression offers a novel strategy to improve treatment efficacy and address drug resistance. Specifically, QRT's integration into combination therapies could amplify therapeutic outcomes by targeting multiple pathways simultaneously [140]. Future research should prioritize identifying and characterizing the miRNA profiles regulated by QRT in different cancer types to uncover specific mechanisms and optimize therapeutic interventions [141]. Furthermore, the development of advanced delivery systems, such as nanoparticles or niosomes, can significantly enhance QRT's bioavailability and enable precise miRNA modulation, maximizing its therapeutic potential [135]. To support these insights, Fig. 2 summarizes QRT's mechanisms of action, while Table 2 highlights promising applications of QRT in cancer treatment.

**Table 2:** Overview of *in vitro* and *in vivo* studies highlighting the promising therapeutic efficacy of various QRT formulations against different cancers.

**Model**	**Categories of Cancer**	**Form of QRT**	**Cell Line**	**Animal Model**	**Mechanism**	**Ref.**
***In vitro*** ***In vivo***	Skin cancer	Titanium dioxide nanotubes conjugated with QRT	B16F10 murine melanoma	Mice	Inhibited tumor growth by regulating phospho-STAT3 levels in the tumor microenvironment and significantly inhibited the blood vessel formation in chick chorioallantoic membrane assay	[144]
***In vitro*** ***In vivo***	Gastric cancer	Pure	Gastric cancer cell lines (AGS, MKN45, MKN7 and TMK1)	Mice	QRT significantly inhibited cell viability and tumor volume compared to the control group. Additionally, QRT was found to decrease glutathione (GSH), malondialdehyde, and reactive oxygen species (ROS) levels while suppressing beclin1 and LC3B levels in cancer cells.	[145]
***In vivo***	Hepatic cancer	Pure	–	Mice	QRT can significantly inhibit HepG2 cell proliferation through the regulation of cyclin D1 expression	[146]
***In vitro*** ***In vivo***	Gastric cancer	QRT combined with irinotecan/SN-38	AGS human gastric adenocarcinoma	Mice	QRT was capable of improving the efficacy of the irinotecan metabolite, SN-38; it ameliorated p-GSK-3βSer9 and β-catenin protein expression levels that were up-regulated by SN-38. It was also found that the combination of QRT and irinotecan had superior modulation of angiogenesis-associated and EMT-related factors.	[147]
***In vitro*** ***In vivo***	Lung cancer	Pure	Lung cancer cell lines (A549 e H69)	Rats	QRT induced apoptosis in human lung cancer cells and showed ability in supressing oxidative stress by reducing MDA and increase of antioxidant enzymes (SOD and GSHP). It also played QRT has played a therapeutic role as it has improved restoring of the damaged lung tissue.	[148]
***In vitro***	Bladder cancer	QRT–zinc complex	BFTC-905 cells	–	Both the cell migratability and invasiveness were markedly reduced by the complex through p-AKT and MT1-MMP regulations.	[149]
***In vitro***	Breast Cancer	QRT-3-D-xyloside	Breast cancer cells (CRL-4010, MCF7 and MDA-MB-231 c)	–	It showed to markedly inhibit the cell viability and migration in breast câncer.	[150]
***In vitro***	Human esophageal cancer	Pure	Human esophageal cancer cell line Eca109	–	QRT suppressed the invasion and angiogenesis of esophageal cancer cells by decreasing expression of VEGF-A, MMP2, and MMP9 proteins.	[151]
***In vitro***	Collon cancer	Pure	Human collon cancer cells lines (Colo-320 and Colo-741)	–	QRT induced apoptosis in primary cancer cells and also triggered secescence in some collon cancer cells.	[152]
***In vitro*** ***In vivo***	Collon cancer	QRT + ionizing radiation	Human colon cancer cell DLD-1	Mice	The combination of a pretreatment with QRT with low doses of ionizing radiation inhibits colon câncer cells by targeting the Notch-1 signaling.	[153]
***In vitro*** ***In vivo***	Lung cancer	Pure/QRT + brigatinib	H1975-MS35 cells carrying EGFR C797S mutation	Mice	The EGFR C797S mutation is one of the known acquired-resistance mutations to the latest third generation of tyrosine kinase inhibitors used to treat non-small-cell lung câncer (NCCLC). In this study, QRT inhibited the tumor growth of xenografted NSCLC cells harboring the EGFR C797S mutation. It also showed sinergistically activuty with brigatinib to inhibit tumor growth *in vivo*.	[154]
***In vitro***	Oral cancer	Pure	Human oral cancer cell lines (HSC-6 and SCC-9)	–	QRT inhibited cell viability, migration and invasion by regulating microRNA-16 and homeobox A10 in oral cancer cells	[155]
***In vivo***	Collon cancer	Pure	–	Rats	QRT activity lead to increase in apoptotic proteins gene expression including caspase 3 and decrease in anti-apoptotic gene expression including Bcl-2 in collon cancer cells,	[156]
***In vitro***	Breast cancer	QRT-loaded spanlastics + letrozole	Human breast cancer cell lines MCF-7	–	This combination was superior to the individual treatments and the soluble free drugs in terms of *in vitro* cytotoxicity for breast cancer cells.	[157]
***In vitro***	Neuroblastoma	QRT loaded chitosan nanoparticles	SH-SY5Y and NIH 3T3 cells	–	QRT nanoparticles reduced cell viability in SH-SY5Y cells at diferent concentrations and it signifcantly enhanced the levels of 8-oxo-dG, cleaved caspase 3, Bax, cleaved PARP, oxidant stress, DNA damage, and eventually apoptose.	[158]
***In vitro***	Breast cancer	QRT + Docetaxel	Human breast cancer cell lines MCF-7	–	These two molecules worked in a synergetic manner, where QRT decreased the expression of Lef1 and resensitized cells to docetaxel, reducing the viability of Docetaxel-resistant cancer cells.	[159]

**Fig. 3:**
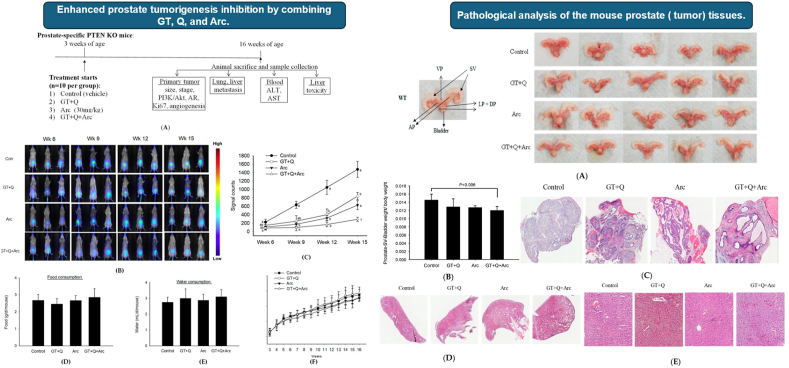
**(Left)** Effect of green tea (GT), QRT (Q), and arctigenin (Arc) on prostate tumorigenesis: (A) Study design. (B) *In vivo* imaging shows tumor inhibition, most notable in the GT + Q + Arc group. (C) Quantification of tumor signal intensity. (D–F) No differences in food, water intake, or body weight were observed. **(Right)** Pathological analysis: (A) Representative prostate images show reduced tumor size in GT + Q + Arc. (B) Prostate weight normalized to body weight. (C) H&E staining reveals reduced tumor grade in treatment groups, especially GT + Q + Arc. No metastasis was detected. Reprinted/adapted with permission from Hao et al. [143]. Copyright 2024, Biomolecules from MDPI (Open Access).

Recent studies on the application of QRT *in vivo* demonstrate its efficacy in cancer treatment, as illustrated in Fig. 3. In Hao et al. [143], QRT's ability to modulate key molecular and epigenetic pathways, such as the PI3K/Akt signaling pathway and microRNA regulation, has been shown to inhibit tumorigenesis and reduce cancer progression. Its synergistic effects when combined with other natural compounds, like arctigenin and green tea catechins, further enhance its chemopreventive potential. For instance, *in vivo* studies using prostate-specific PTEN knockout mice have revealed that QRT, when combined with green tea and arctigenin, significantly inhibits tumorigenesis by 90 %, compared to single treatments. This combination not only reduced tumor size but also limited progression to lower-grade lesions, as evidenced by histopathological examination. These effects are attributed to the suppression of androgen receptor activity, decreased proliferation markers (Ki67), and inhibition of angiogenesis.

## 6 Improving the stability of quercetin with drug delivery systems

Certain technologies facilitate the administration of bioactive compounds that are typically unstable during therapeutic use, enabling precise dose control and minimizing side effects. As noted earlier, antioxidants can serve as effective agents against various cancers, however, a range of environmental factors (light, temperature, pH) and conditions within biological systems (like hepatic metabolism, oxidative reactions and interactions with the microbiota) can destabilize these compounds and diminish their anticancer effects [160,161]. Below are the most used encapsulation methods in the food industry, which are also industrially scalable.

In the food and pharmaceutical industries, some easier and less costly techniques have gained relevance [56]. Among these methods, one of the most accepted is ionic gelation; ionic gelation involves encapsulating compounds through ion exchange in dispersive systems. The most commonly used studies of this method are based on alginate and chitosan, as alginate presents a net negative ionic charge, while chitosan has a net positive charge [162]. Both polymers seek an exchange with an ion of the opposite charge to successfully recirculate. For this purpose, chloride salts (Ca^+2^, Al^+3^) and tripolyphosphate (TPP-) are considered excellent options since capsule formation (micro or nano) occurs instantly (in most cases) or with external induction mechanisms such as ultrasound [163,164]. Spray drying is a widely used technique for protecting active ingredients and heat-sensitive compounds, serving both as a dehydration and encapsulation method. It transforms a liquid feed, such as an emulsion or solution, into a powder by spraying it into a hot drying gas; the process begins with the liquid being atomized into small droplets as it enters the drying chamber, where it contacts heated air or an inert gas, leading to particle development through nucleation, growth, and later agglomeration while the particles move in a helical motion, aided by friction forces that create a smooth, spherical morphology, ultimately resulting in the dry product being collected at the bottom of the chamber [165].

Encapsulation by Coacervation is based in a phase separation process that occurs in a liquid medium when specific physico-chemical conditions, such as pH, ionic strength, and temperature, are controlled, resulting in two distinct phases: a polymer-rich coacervate and a diluted supernatant. In complex coacervation, oppositely charged polyelectrolytes form an insoluble macromolecule complex through electrostatic interactions, which can occur reversibly with changes in temperature or pH, creating amorphous liquid droplets that can separate from the water by gravity; this process is cost-effective and operates under mild conditions, utilizing widely available biodegradable and food-grade macromolecules [166].

Fluidized bed encapsulation enables the coating and enteric coating of bioactive compounds. In this type of encapsulation, air flow, vibration, flow, and temperature conditions must be managed and it generally protects compounds sensitive to gastrointestinal pH [167]. A clear example of its application is hydroxypropyl methylcellulose acetate succinate, which has gastroprotective properties, preventing the release of acid-labile/unstable compounds in the stomach but releasing them in the intestine, with solubility ranging from pH 6.5 to 7.4 [168]. Inclusion complexation is a method based on combining a compound that acts as a ligand, which is the bioactive compound (generally volatile such as essential oils and vitamins), and the porous or coating material (such as β-cyclodextrins and β-lactoglobulin) that bind through hydrophobic entropic impulses, including ionic forces, hydrogen bonds, and van der Waals forces [169].

The nanoencapsulation process involves enclosing solid, liquid, or gaseous substances within tiny containers known as capsules, typically surrounded by a coating material that acts as a protective barrier against environmental factors and chemical interactions; these capsules can be produced at the nanoscale (<100 nm) using various materials for the membrane, often incorporating bio-based components [170,171]. Among these compounds are carbohydrates like starch, maltodextrins, corn syrup solids, dextran, modified starch, sucrose, cyclodextrins, and marine carbohydrates, along with gum derivatives such as gum arabic, agar, sodium alginate, and carrageenan; lipid derivatives include wax, paraffin, beeswax, and various glycerides [208]. Additionally, proteins like gluten, casein, gelatin, albumin, and hemoglobin can be modified for bioactive transport, while cellulose derivatives such as carboxymethyl cellulose and methylcellulose serve as effective drug protectors and potential coatings [172].

QRT has significant difficulty due to its low solubility in aqueous solutions, which limits its application in the food and pharmaceutical sectors [173]. Several studies have demonstrated that its encapsulation in different nano/microsystems can improve its administration and transport (Table 3); recently, the use of organometallic capsules with γ-cyclodextrin was reported to enhance the absorption and effective targeting of QRT. These did not produce cytotoxicity in proximal tubular renal cells HK-2 but inhibited the proliferation of a colon cancer cell line HT-29 [173].

**Table 3:** Important applications of QRT nanoencapsulated systems against cancer cells.

**Encapsulation**	**Highlights of Anticancer Activity**	**Ref.**
Polymeric - psyllium crosslinked pH-sensitive grafted-poly(acrylonitrile-co-acrylic acid)	Colon - showed maximum swelling at pH 7 and demonstrated maximum release of QRT nanoparticles (93 %) at the intestinal fluid pH (pH 7.4).	[174]
Vesicles of hydrogenated phosphatidylcholine from soy origin, content of 90 % + scorpion venom + QRT	Breast - significant cell cycle arrest in the S phase, increased mRNA expression of caspase-9, Bax, Bcl-2, and p53; and reduced TNF-α and NF-κB activity.	[175]
Monolayer and bilayer emulsions of soy protein isolate	Chitosan assembly in the emulsion can enhance QRT's bioavailability. Succinylation prolongs the digestion time of the emulsion in the small intestine.	[176]
Ethanol nanoemulsions + tween 80	The nanoemulsion was the most suitable for QRT release, and both the nanoemulsion and the emulsion gel exhibited the highest bioaccessibilities.	[177]
Poly(d,l)-lactic-co-glycolic acid (PLGA) nanocapsules	Activity against breast cancer cell lines CAL51 and MCF7 was evaluated using DNA fragmentation assays, fluorescence microscopy, and double staining with acridine orange and propidium iodide, exhibiting apoptotic activity.	[178]
PLGA nanoparticles + caffeic acid phenethyl ester	Increased mRNA levels of caspase-3 (2.38-fold) and caspase-9 (2-fold) and key protein expressions in the intrinsic apoptosis pathway in colon cancer cells HT-29.	[179]
Agarose-polyvinylpyrrolidone-hydroxyapatite hydrogel-loaded nanoemulsion	pH-sensitive nanocarrier for controlled QRT release in MCF-7 breast cancer cells.	[180]
Hybrid polymer-lipid nanoparticles + zinc phthalocyanine (photosensitizer)	The photodynamic effect of the photosensitizer was synergized by QRT, increasing anticancer activity, indicating great potential for future cancer treatment and mitigating chemotherapy side effects.	[181]
Alginate nanoparticles	*In vitro* drug release showed sustained QRT release for up to 6 days against human leukemic U937 cancer cells.	[182]
Solid lipid nanoparticles loaded with Erlotinib and QRT + chitosan	Demonstrated significant clinical improvement against non-small cell lung cancer (A549 and NCI H460 cells) and evidenced a synergistic effect with QRT, reducing adverse effects.	[183]
CuO nanoparticles functionalized with chitosan encapsulated in QRT	*In vitro* studies demonstrate potent anticancer activities against MCF-7 cells, reduction in breast tumour volume in female rats induced by dimethylbenz(a)anthracene; induced apoptosis through increased p53 gene and cell cycle arrest. Also, increased cytochrome *c* and caspase-3 levels, leading to breast carcinoma cell death.	[184]
pH-ultrasensitive nanoparticles based on Fe2O3-chitosan-montmorillonite	Analysis showed controlled and targeted QRT release, indicating a 43 % cumulative release during the first 12 h and 65 % cell death (MCF-7), leading to reduced side effects and selective destruction of cancer cells compared to non-targeted drugs.	[185]
Soy/chitosan nanocapsules	Adding chitosan provides a more stable complex, exhibiting antioxidant, anti-inflammatory, and anticancer activities (human colon carcinoma cell lines HCT-116 and human osteosarcoma U2OS). It can also be added to a solution containing water, thus producing a beverage complemented with bioactive and soluble QRT.	[186]
Nanoemulsion	Cisplatin is a potent antitumor agent widely used clinically, especially for treating solid tumors (uterine, testicular, bladder, lung, head, and neck). A synergistic effect of Cisplatin/QRT was demonstrated against highly aggressive and metastatic breast cancer cells (MDA-MB-231).	[187]
β-cyclodextrin microparticles	The absorption of QRT is very low when not encapsulated. In this nanosystem, cyclodextrins encapsulated within a polymeric matrix by spray dryer were used. Its activity against alveolar adenocarcinoma cells was reported, potentially improving bioavailability and activity for a longer time and in a controlled dose.	[188]
Chitosan nanohydrogel	New nanohydrogels with antimicrobial and anticancer activities significantly reduce levels of DNA methyltransferases (DNMT), responsible for DNA methylation in HepG2 cells. Combined therapy in these cases is proposed as an excellent application option.	[189]
Ethyl cellulose/gelatin hybrid nanofibers	Adding hydrophilic gelatin to the system decreased the thermal stability of the nanofibers while improving their surface wettability; these nanofibers also showed good stability in acidic and aqueous food environments, and the inclusion of gelatin enhanced the release of QRT in the colon. The anticancer activity against HCT-116 cells was demonstrated by inducing apoptosis and reducing cell viability.	[190]
Hyaluronic nano-micelles	The hyaluronic acid polymers self-assembles with QRT to form drug-carrying micelles, which exhibited high cytotoxicity and apoptosis-inducing abilities, ascribed to the pH-sensitive micelles accomplishing rapid drug release of QRT under low pH condition. *in vivo* experiments showed that this nanosystem effectively inhibited tumour growth in tumour-bearing mice, prolonged the survival time of this type of tumour and reduced the toxicity of the drug to normal tissues.	[191]
QRT-fucoidan nanoparticles	Fucoidan is a natural polysaccharide derived from marine sources that exhibits immunomodulatory properties. The antitumor effects of this nanosystem operate through several mechanisms, such as inducing oxidative stress in tumour cells, hindering cell cycle progression, and facilitating apoptosis. Additionally, these nanoparticles have been shown to inhibit the proliferation and migration of cancer cells *in vivo*. The treatment with this nanosystem resulted in elevated levels of macrophage surface markers, indicating that QRT-fucoidan nanoparticles may act as an immunotherapeutic agent by enhancing macrophage activation.	[192]
Polyamidoamine dendrimer decorated graphene oxide	The poly(amido amine) (PAMAM) dendrimer decorated graphene oxide (GO) shows higher QRT loading capacity compared to GO alone. When loaded with QRT, it exhibits a pH-responsive release behaviour and the amount of QRT released at pH 4 is higher than the release at pH 7.4; moreover, it exhibits higher cytotoxic effects than pure QRT towards human breast cancer MDA MB 231 cells.	[193]

**Fig. 4:**
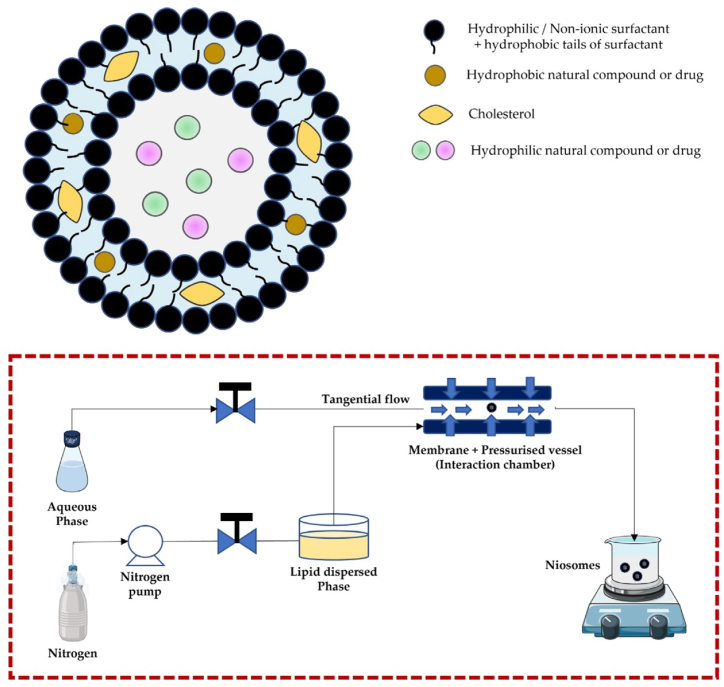
Schematic representation of niosome encapsulation and preparation process. (Top) Structural diagram of a niosome showing the arrangement of hydrophilic and hydrophobic compounds within the bilayer membrane composed of non-ionic surfactants and cholesterol. Hydrophilic compounds are encapsulated in the aqueous core, while hydrophobic compounds are integrated within the lipid bilayer. (Bottom) Process flow for niosome preparation using tangential flow and high-pressure homogenization. The aqueous phase and lipid dispersed phase are combined under controlled conditions using nitrogen pressure, followed by tangential flow filtration through a pressurized vessel to form niosomes. Reprinted/adapted with permission from Liga et al. [196]. Copyright 2024, Pharmaceutics from MDPI.

In recent years, niosomes have gained attention as an effective delivery system for bioactive compounds like QRT. These are vesicles made from non-ionic surfactants with a bilayer structure, which provide excellent encapsulation efficiency for hydrophobic compounds while safeguarding them against environmental and physiological degradation [194]. Niosomes improve the stability, bioavailability, and therapeutic impact of QRT by controlling its release and reducing its interactions with metabolic enzymes or oxidative agents [195]. Common methods for preparing niosomes include thin-film hydration, reverse-phase evaporation, and microfluidics, which help create uniform vesicles. Their composition, typically a combination of non-ionic surfactants, cholesterol, and sometimes stabilizers, can be tailored to meet specific therapeutic needs (Fig. 4) [196]. For example, ligands such as folic acid or hyaluronic acid can be added to niosomes for targeted delivery to cancer cells that overexpress folate or CD44 receptors, respectively [197,198]. In addition, surface modifications can be made through covalent or electrostatic interactions, which enhance the selectivity and effectiveness of QRT delivery [199]. This ability to target specific cells not only boosts QRT's anticancer properties but also minimizes side effects, making niosomes an attractive option for cancer treatment [200,201].

**Fig. 5:**
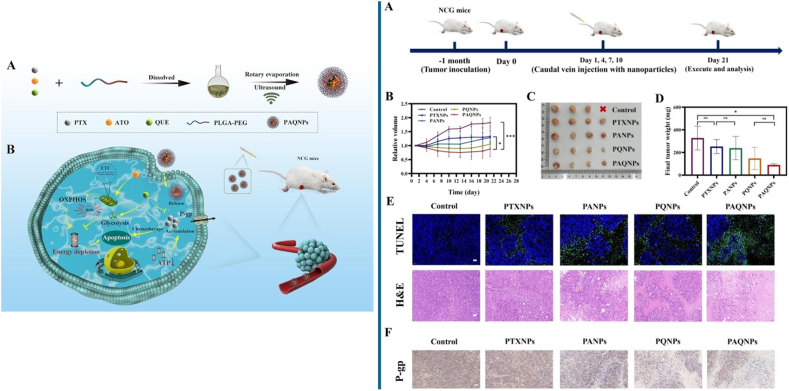
**(Left)** (A) Preparation process of PAQNPs. (B) Mechanism of PAQNPs in regulating energy metabolism to reverse multidrug resistance in ovarian cancer. **(Right)** (A) Treatment protocol for the A2780/Taxol tumor-bearing NCG mice model. (B) Tumor volume progression under different treatments. (C) Tumor images post-dissection. (D) Final tumor weights. (E) TUNEL and H&E staining of tumor tissues. (F) IHC analysis of P-gp expression in tumors. Scale bars: 50 μm. Reprinted/adapted with permission from Lu et al. [202]. Copyright 2024, International Journal of Pharmaceutics from ElSevier.

A recent study involving nanoparticles incorporating QRT has demonstrated a promising approach to overcoming multidrug resistance (MDR) in ovarian cancer treatment [202]. These nanoparticles, termed PTX-ATO-QUE (PAQNPs), utilize a PLGA-PEG platform to co-deliver atovaquone (ATO), an inhibitor of mitochondrial oxidative phosphorylation (OXPHOS); QRT, a glycolysis inhibitor; and paclitaxel (PTX), a chemotherapeutic drug. PAQNPs act by inhibiting OXPHOS and glycolysis pathways through suppression of mitochondrial complex III and hexokinase II (HK II) activity, thereby reducing intracellular ATP levels and P-gp activity, which increases PTX accumulation. This, combined with elevated reactive oxygen species (ROS) levels, induces apoptosis in chemotherapy-resistant cells. Validated *in vivo*, PAQNPs significantly inhibited tumor growth in mice and showed a favorable safety profile, highlighting their potential to reverse MDR and enhance chemotherapy efficacy in ovarian cancer (Fig. 5).[210]

## 7 Perspectives and clinical studies

QRT's role in cancer therapy has expanded significantly, as recent clinical studies explore its potential in targeting various cancer types. Notably, QRT has shown promise in enhancing immune response and inducing apoptosis in multiple cancers, particularly when used in combination with other treatments [18]. For example, a recent clinical study tested the combination of QRT and dasatinib to reduce cellular senescence, particularly in adult survivors of childhood cancers (NCT04733534). This approach capitalizes on QRT's senolytic activity, targeting senescent cells that contribute to cancer relapse and overall patient frailty. In prostate cancer research, QRT has been investigated for its effects in conjunction with green tea polyphenols to optimize uptake in prostate tissue, especially in patients scheduled for surgery (NCT01912820). This type of co-administration could potentially improve the bioavailability of active compounds within tumour tissues, enhancing therapeutic efficacy against prostate malignancies. For breast cancer, QRT has been studied in the context of immunotherapy by modulating the JAK/STAT1 pathway where QRT can inhibit tumour immune escape mechanisms, thereby improving T cell recognition and response against tumour cells. This effect, particularly against triple-negative breast cancer, offers new avenues for immune-based therapies [203].

Furthermore, new trials are also assessing nanoformulations of QRT, which allow for targeted drug delivery to cancer cells. This nanotechnology approach may not only increase QRT's stability and bioavailability but also enhance its selectivity for tumour cells, particularly glioblastoma, where targeting is a key challenge [204]. For instance, QRT's conjugation with nanoparticles aims to improve tumour-specific apoptosis and reduce systemic side effects often associated with conventional therapies. These findings suggest that future research should focus on combining QRT with both immune-modulating agents and nanotechnology-based delivery systems, potentially paving the way for more effective treatments of challenging cancers like glioblastoma, prostate, and breast cancer.

## 8 Conclusions

Flavonoids possess potential anticancer activity, with most studies focusing on QRT, which has demonstrated its effectiveness and selectivity in *in vitro* and *in vivo* studies, as well as reaching its clinical phase. QRT and other flavonoids achieve greater specificity towards cancer cells through targeted delivery by nanoparticles, drastically increasing their bioavailability, stability, and half-life. It is concluded that in the coming years, new drugs (either standalone or combined) will include many flavonoids for the treatment and prevention of various types of cancer.

## CRediT authorship contribution statement

**Piero Alex Silva-Pinto:** Formal analysis, Investigation, Methodology, Writing – original draft. **Janaína Teixeira Costa de Pontes:** Investigation, Methodology, Writing – original draft. **Brigitte Aguilar-Morón:** Investigation, Methodology. **Christian Shleider Carnero Canales:** Supervision, Validation, Visualization. **Fernando Rogério Pavan:** Supervision, Validation, Visualization. **Cesar Augusto Roque-Borda:** Conceptualization, Investigation, Methodology, Supervision, Validation, Visualization, Writing – review & editing.

## Declaration of competing interest

All contributing authors declare no conflicts of interest.
